# A fascia lata free flap in pelvic exenteration for Fournier gangrene due to advanced rectal cancer: a case report

**DOI:** 10.1186/s40792-017-0350-y

**Published:** 2017-05-26

**Authors:** Hiroshi Sawayama, Nobutomo Miyanari, Hidetaka Sugihara, Shiro Iwagami, Takao Mizumoto, Tatsuo Kubota, Yoshio Haga, Hideo Baba

**Affiliations:** 1 Department of Surgery, National Hospital Organization Kumamoto Medical Center, 1-5 Ninomaru, Kumamoto, 860-0008 Japan; 2 Department of Gastroenterological Surgery, Graduate School of Medical Sciences, Kumamoto University, 1-1-1 Honjo, Kumamoto, 860-8556 Japan

**Keywords:** Fascia lata, Pelvic exenteration, Fournier gangrene, Rectal cancer

## Abstract

**Background:**

Fournier gangrene due to advanced rectal cancer is a rapidly progressive gangrene of the perineum and buttocks. Emergency surgical debridement of necrotic tissue is crucial, and secondary surgery to resect tumors is necessary for wound healing. However, pelvic exenteration damages the pelvic floor, increasing the likelihood of herniation of internal organs into the infectious wound. The management of pelvic exenteration for rectal cancer with Fournier gangrene has not yet been established. We herein describe the use of a fascia lata free flap in pelvic exenteration for rectal cancer with Fournier gangrene.

**Case presentation:**

A 66-year-old male who had undergone colostomy for large bowel obstruction due to advanced rectal cancer and continued chemotherapy was referred to our hospital for Fournier gangrene resulting from chemotherapy. Emergency surgical debridement was performed, and the infectious wound around the rectal cancer was treated with intravenous antibiotic agents postoperatively. However, the tumor was exposed by the wound, and exudate persisted. Pelvic exenteration was performed due to tumor infiltration into the bladder and prostate. Tumor resection resulted in a defect in the pelvic floor. A fascia lata free flap (15 × 9 cm) obtained from the left thigh was fixed to the edge of the peritoneum and ileal conduit to close the defect in the pelvic floor and prevent small bowel herniation into the resected space. There was no intraabdominal inflammation or bowel obstruction postoperatively, and outpatient chemotherapy was continued.

**Conclusions:**

Surgical repair with a fascia lata free flap to close the defect in the pelvic floor led to a good clinical outcome for pelvic exenteration in a patient with Fournier gangrene due to advanced rectal cancer.

## Background

Prosthetic material is contraindicated for infected or contaminated abdominal wall defects; hence, the repair of such defects is challenging [1, 2]. Infected incisional hernias have been treated using autologous tissue grafts, and a previous study reported that treatment with a fascia lata patch is safe and effective [3].

Fournier gangrene is a rapidly progressive condition in which polymicrobial necrotizing fasciitis develops in the perineal, perianal, or genital areas [4]. Surgical debridement of necrotic tissue is crucial, and tumor excision is necessary for wound healing. The management of pelvic exenteration for advanced rectal cancer with Fournier gangrene has not yet been established.

We herein describe the application of a fascia lata free flap to prevent internal organs herniating into the infectious wound following pelvic exenteration for Fournier gangrene due to advanced rectal cancer.

## Case presentation

**Fig. 1:**
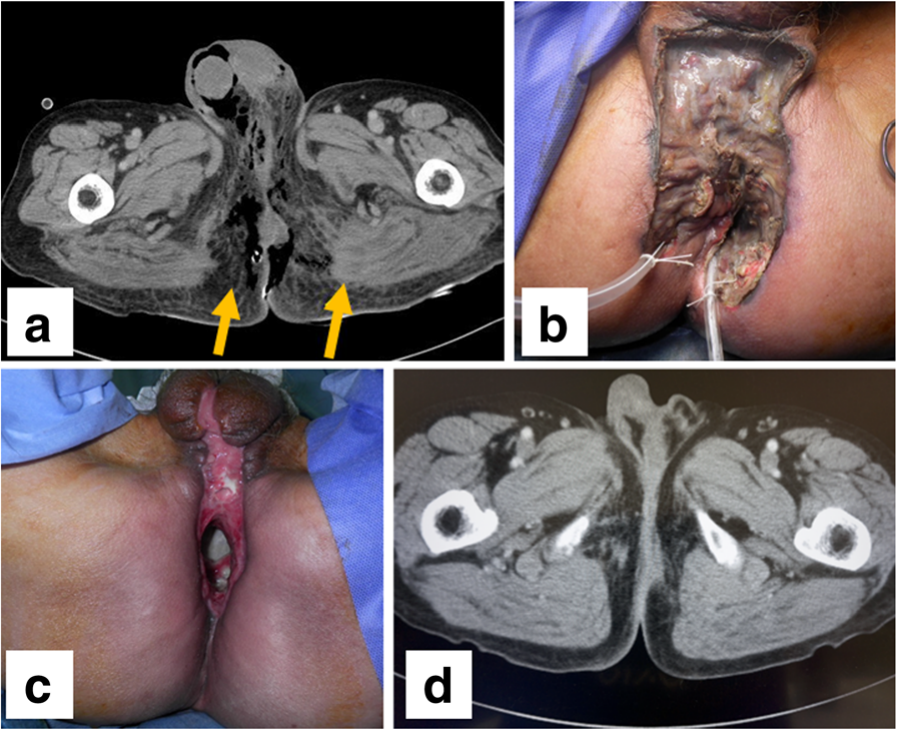
Computed tomography and pictures of the perineum and buttocks region. Computed tomography showed necrotizing soft tissue infection with large amounts of gas throughout the perineum (*arrows*) (**a**). The necrotic soft tissue in the perineum and buttocks surrounding rectal cancer was resected (**b**). The perineum and buttocks region on day 35 after total pelvic exenteration (**c**). Computed tomography 8 months postoperatively (**d**)

A 66-year-old male underwent colostomy for large bowel obstruction due to advanced rectal cancer and continued chemotherapy. The patient was referred to our hospital for Fournier gangrene due to the penetration of the advanced rectal cancer during chemotherapy. The perineum and buttocks around the anus exhibited swelling and skin necrosis with severe pain and a septic odor. Laboratory testing showed leukocytosis (11,500/μl) and elevated C-reactive protein (38.94 mg/dl). Abdominal computed tomography revealed a necrotizing soft tissue infection with large amounts of gas throughout the perineum (Fig. 1).

**Fig. 2:**
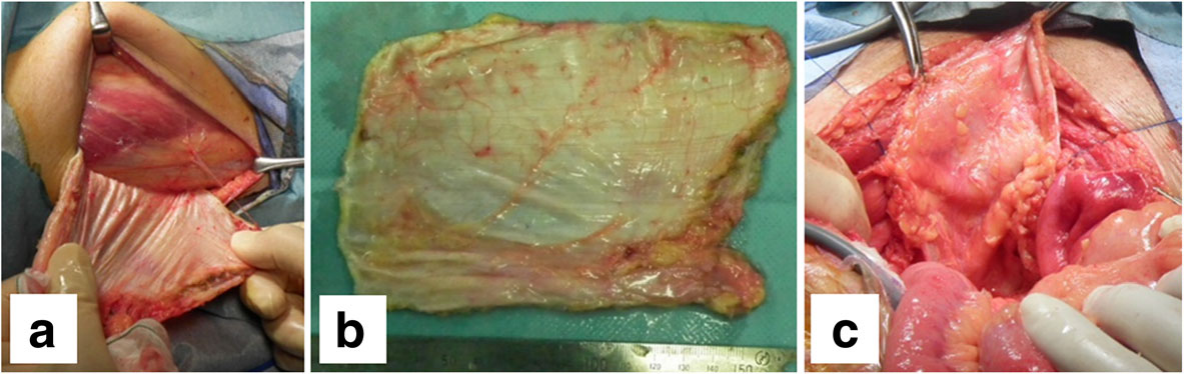
Surgical repair of the pelvic floor defect with a fascia lata free flap. A 15-cm incision was made, and a fascia lata flap (15 × 9 cm) was created from the left thigh (**a**, **b**). The patch was fixed to the edge of peritoneum and the ileal conduit wall (**c**)

Emergency surgical debridement was performed on the areas of the perineum and buttocks surrounding the rectal cancer (Fig. 2). The infected wound around the rectal cancer was washed, and intravenous antibiotics were administered; however, the tumor was exposed by the wound, and exudate persisted. Tumor resection was considered necessary to treat the infectious wound, and radical surgery was performed on day 15 after debridement surgery. General anesthesia was induced, and a skin incision was made in the lower abdomen. Pelvic exenteration was performed due to the infiltration of the tumor into the bladder and prostate. Tumor resection resulted in a large defect in the pelvic floor, increasing the likelihood of internal organs herniating into the infectious wound. A colostomy was constructed through the left rectus abdominis muscle, and an ileal conduit was constructed through the right rectus abdominis muscle. The omentum was adhered and atrophied, due to previous surgery, and was not sufficient to fill the large pelvic defect.

A 15-cm incision was made and a fascia lata free flap (15 × 9 cm) was created from the left femur (Fig. 2a, b). The ventral, lateral, and back sides of the flap were fixed to the edge of the peritoneum along the resected bladder, bilateral external iliac arteries, and ileal conduit, respectively, (Fig. 2c). After the rectum was resected, the pelvic space was left and the perineal wound was not sutured. However, the perineal wound was separated from the abdominal cavity.

Histological examination revealed moderately differentiated tubular adenocarcinoma of the rectum with direct invasion of the prostate and vas deferens. Severe lymphatic and vascular invasion were present, and four metastases of the regional lymph nodes were identified. A swollen right inguinal lymph node was dissected and diagnosed as a metastasis from rectal cancer. The tumor was diagnosed as stage IV, pT4b (prostate and vas deferens) N2 M1 (inguinal lymph node metastasis) according to the Japanese Classification of Colorectal Carcinoma. The tumor was resected with negative proximal and distal margins; however, this case had a high risk of local recurrence, and so, postoperative chemotherapy and chemoradiotherapy were planned.

**Fig. 3:**
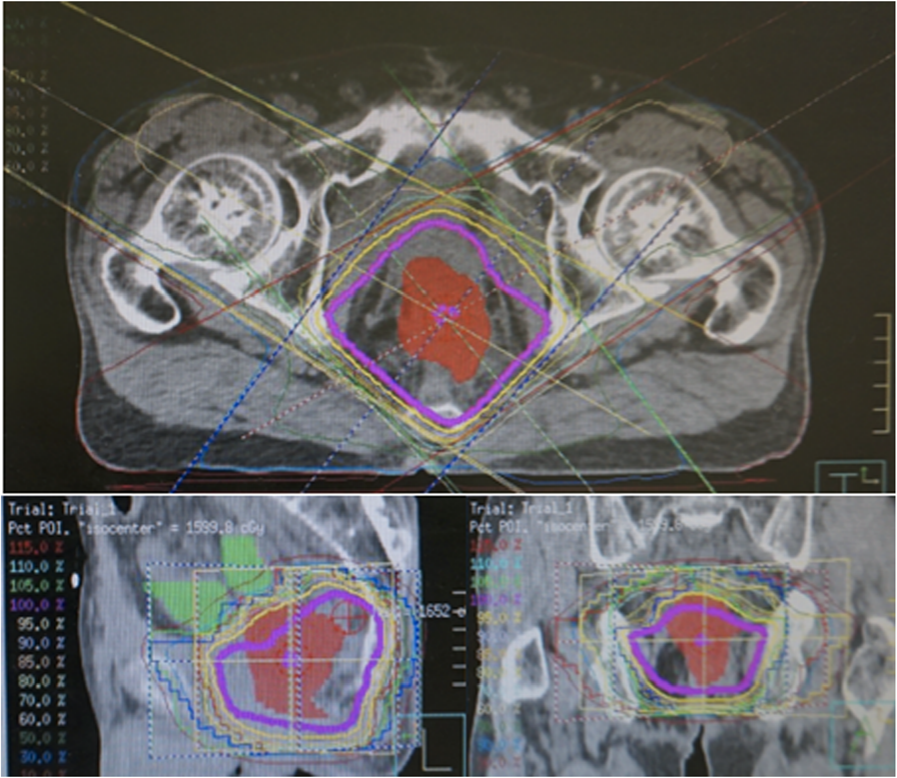
Irradiation plan for the pelvic area

The patient ceased fasting on postoperative day 2. There was no postoperative intraabdominal inflammation or bowel obstruction. The perineal wound was managed as an open wound. The wound exudate became clear, and the wound was considered to be healed on postoperative day 35 (Fig. 1c). Postoperative computed tomography showed maintenance of the fascia lata free flap. There was no weakness in the left leg, and the patient maintained the same walking ability as preoperatively. Outpatient chemotherapy was continued after the patient was discharged from hospital. Radiotherapy of the pelvic area was performed postoperatively. Irradiation of the pelvic space was performed, while minimizing irradiation of the small bowel (Fig. 3). The pelvic space had disappeared by 8 months postoperatively (Fig. 1d). Positron emission tomography performed 1.5 years postoperatively showed no accumulation of 18F-fluoro-deoxy-glucose in the pelvic area. The patient has had no further complications in 2 years of follow-up.

## Discussion

The treatment of Fournier gangrene due to advanced rectal cancer is challenging, as tumor resection is required to remove the infectious area. Pelvic exenteration results in a large defect in the pelvic floor, increasing the likelihood of internal organs herniating into the infectious wound. In the present case, this defect was safely and effectively repaired with a fascia lata free flap.

Fournier gangrene is characterized by rapidly progressive necrotizing fasciitis in the perineum and external genital organs. The sources and etiological factors of Fournier gangrene are dermatological (25%), anorectal (21%), diabetes mellitus (20%), urological (19%), and alcohol abuse (9%) [5]. Only a limited number of studies have reported spontaneous perforation of rectal cancer presenting as Fournier gangrene of the perineum and scrotum [6–9]. Fournier gangrene is a true emergency that requires aggressive treatment with antibiotics and immediate surgical debridement [10]. Although our patient underwent emergency surgical debridement, the tumor was exposed by the wound, and exudate persisted. We considered that tumor excision was essential to enable adequate wound treatment; this tumor excision resulted in a large defect in the pelvic floor.

The standard surgical procedure used to treat abdominal wall defects is mesh repair [11, 12]. However, mesh repair is contraindicated in infected abdominal wall defects. A number of studies have reported the repair of an abdominal wall defect using a fascia lata patch [3, 13, 14]. In the present case, we used a fascia lata free flap to close the pelvic floor defect. The advantages of a free fascia lata flap are that only a short period of time is required to create the flap, the method is technically simple, leg strength is minimally affected, and there are multiple locations to which the flap can be fixed.

There may be an additional advantage to using a fascia lata free flap in pelvic exenteration with Fournier gangrene. The small bowel enters the irradiation field during radiation therapy for rectal cancer, which increases the risk of adverse effects [15]. Using a fascia lata free flap to close, the pelvic floor defect ensures that the small bowel no longer drops into the pelvic space, which may reduce the adverse effects associated with postoperative radiation therapy for pelvic lesions.

## Conclusions

We describe a case in which the patient underwent pelvic exenteration for Fournier gangrene due to advanced rectal cancer. Surgical repair of the pelvic floor defect with a fascia lata free flap led to a good clinical course and prevented herniation of internal organs into the infectious wound.

## References

[1] Yang L, Wang H, Liang X, Chen T, Chen W, Song Y (2015). Bacteria in hernia sac: an important risk fact for surgical site infection after incarcerated hernia repair. Hernia.

[2] Sawayama H, Kanemitsu K, Okuma T, Inoue K, Yamamoto K, Baba H (2014). Safety of polypropylene mesh for incarcerated groin and obturator hernias: a retrospective study of 110 patients. Hernia.

[3] Miyamoto Y, Watanabe M, Ishimoto T, Baba Y, Iwagami S, Sakamoto Y (2015). Fascia lata onlay patch for repairing infected incisional hernias. Surg Today.

[4] Shyam DC, Rapsang AG (2013). Fournier’s gangrene. Surgeon.

[5] Eke N (2000). Fournier’s gangrene: a review of 1726 cases. Br J Surg.

[6] Ossibi PE, Souiki T, Ibn Majdoub K, Toughrai I, Laalim SA, Mazaz K (2015). Fournier gangrene: rare complication of rectal cancer. Pan Afr Med J.

[7] Carr JA (2010). Perforated rectal cancer presenting as Fournier’s gangrene. J Clin Oncol.

[8] Gamboa EO, Rehmus EH, Haller N (2010). Fournier’s gangrene as a possible side effect of bevacizumab therapy for resected colorectal cancer. Clin Colorectal Cancer.

[9] Rajendran S, Khan A, Murphy M, O’Hanlon D. Rectocutaneous fistula with Fournier’s gangrene, a rare presentation of rectal cancer. BMJ case reports. 2011;2011. doi:10.1136/bcr.06.2011.4372. 10.1136/bcr.06.2011.4372 PMC3149482 22689729

[10] Ash L, Hale J (2005). CT findings of perforated rectal carcinoma presenting as Fournier’s gangrene in the emergency department. Emerg Radiol.

[11] Luijendijk RW, Hop WC, van den Tol MP, de Lange DC, Braaksma MM JNIJ (2000). A comparison of suture repair with mesh repair for incisional hernia. N Engl J Med.

[12] Kingsnorth A, LeBlanc K (2003). Hernias: inguinal and incisional. Lancet.

[13] Lv Y, Cao D, Guo F, Qian Y, Wang C, Wang D (2015). Abdominal wall reconstruction using a combination of free tensor fasciae lata and anterolateral thigh myocutaneous flap: a prospective study in 16 patients. Am J Surg.

[14] Li EN, Silverman RP, Goldberg NH (2005). Incisional hernia repair in renal transplantation patients. Hernia.

[15] Wang BL, Jiang W, Du SS, Xu JM, Zeng ZC (2012). The therapeutic and adverse effects of modified radiation fields for patients with rectal cancer. Clin Colorectal Cancer.

